# Individualized Efficiency of Traditional Chinese Medicine for Non-ST Segment Elevation Acute Coronary Syndrome: Study Protocol for Observational Research by the Evidence-Based Goal Attainment Scale

**DOI:** 10.1155/2020/7653040

**Published:** 2020-09-14

**Authors:** Zhaofeng Shi, Chen Zhao, Manke Guan, Xuxu Wei, Jiayuan Hu, Xiaoyu Zhang, Min Li, Hongcai Shang

**Affiliations:** ^1^Dongzhimen Hospital, Beijing University of Chinese Medicine, Beijing 100700, China; ^2^Institute of Basic Research in Clinical Medicine, China Academy of Chinese Medical Sciences, Beijing 100700, China; ^3^Beijing Hospital of Traditional Chinese Medicine, Capital Medical University, Beijing 100010, China; ^4^The Third Affiliated Hospital, Beijing University of Chinese Medicine, Beijing 100029, China; ^5^International Evidence-Based Research Institute of Chinese Medicine, Beijing University of Chinese Medicine, Beijing 100029, China

## Abstract

**Background:**

Non-ST segment elevation acute coronary syndrome has been one of the most serious diseases threatening human health. Long-term cardiac rehabilitation and secondary prevention is the essential method to control the recurrence and mortality of the disease. Traditional Chinese medicine has proved the efficiency on the treatment of non-ST segment elevation acute coronary syndrome, but there is a lack of appropriate methodological design to reflect the characteristics of individualized diagnosis and treatment of it. Therefore, this study used the evidenced-based Goal Attainment Scale to evaluate the clinical effectiveness of traditional Chinese medicine on the treatment of non-ST segment elevation acute coronary syndrome.

**Method:**

This is observational research with the prospective feature. A total of 200 patients will be recruited and observed in the three months by telephone or door visit, collecting the individualized intervention of traditional Chinese medicine and evaluating through the method of evidence-based Goal Attainment Scale. Participants will be included according to the inclusion and exclusion criteria. Any reasons for loss to follow-up and adverse events will be recorded strictly. *Discussion*. The evidence-based Goal Attainment Scale provides a personalized method of evaluation based on the Goal Attainment Scale and combined with evidence-based medicine, which can better reflect the characteristics and superiority of individualized and dynamic intervention for traditional Chinese medicine on the long-term prevention and treatment of non-ST segment elevation acute coronary syndrome than other methods of design. It is of great significance to explore and promote this method of design in the future.

## 1. Introduction

Non-ST segment elevation acute coronary syndrome (non-ST-elevation ACS, NSTE-ACS) is a clinical syndrome in which patients have acute chest pain but no sustained ST-segment elevation in electrocardiogram [1]. The clinical spectrum ranges from no symptom to persistent ischemia, instability of electrophysiology or hemodynamics, or cardiac arrest. The incidence of NSTE-ACS has increased in recent years, and the proportion is higher than that of ST-segment elevation acute coronary syndrome (STE-ACS) [2], which constitutes acute coronary syndrome (ACS) with NSTE-ACS together. Since the wide disease spectrum, neuromas complications, and difficult diagnosis in the early stage are the characteristic of NSTE-ACS, positive and effective diagnosis and treatment of it attached great importance in clinical practice [3]. Although the prognosis and long-term survival of patients with NSTE-ACS have improved significantly with the method of early assessment and advance of interventional therapy based on risk stratification and the standardized drug therapy, long-term cardiac rehabilitation and the secondary prevention after the acute phase has become the key to control the recurrence and mortality of cardiac events [4].

Traditional Chinese medicine (TCM) has a history of thousands of years and obtains a unique understanding and methods of therapy for various diseases. According to the clinical manifestations, NSTE-ACS belongs to the category of “Xiong Bi (chest pain)”, “Xin Ji (palpitation),” and “Zhen Xin Tong (pain of the heart)” for TCM's theory [5]. The field of advantage for TCM applied to clinical treatment lies in chronic noncommunicable diseases, which represent that this kind of treatment has certain advantages according to the long-term period individualized intervention. In recent years, the clinical efficacy of TCM, which is categorized as complementary and alternative medicine, has been continuously proved in the treatment of NSTE-ACS [6]. Clinical treatment of TCM belongs to a personalized medical practice model under the guidance of syndrome differentiation, and its clinical efficacy has been verified for thousands of years. However, there is a lack of scientific and standardized methodological design to support the acquisition of individualized evidence of TCM, which is limited in the progress of international promotion and application [7]. It is urgent to establish a method of evaluation for clinical efficacy that conforms to the characteristics of individualized diagnosis and treatment of TCM and in line with current international clinical standards [8].

Goal Attainment Scale (GAS) is a method of evaluation in order to quantify progress toward defined rehabilitation goals [9]. Its principle is to set a number of indicators for specific individuals, calculating the total score and giving each individual the final points through the 5-level Likert scale to evaluate the realization of each index quantificationally. This method could maintain comparability between the individual on the premise of different indexes. Evidence-based GAS is a personalized method of evaluation based on GAS and combined with an evidence-based medicine analysis model, aiming at the individualized trend of clinical evaluation for TCM, which has been highly recognized in China [10]. Both evidence-based GAS and TCM have the same aspect of paying attention to the experience of doctors and the feeling of patients; however, evidence-based GAS is much more objective and convincing than the traditional methodology of TCM for evaluation.

This study protocol was based on the empirical observational research, adopting the method of evidenced-based GAS and collecting the information of diagnosis and treatment about the TCMs individualized therapy of NSTE-ACS, to evaluate the clinical effectiveness and variation of therapy for TCM objectively.

## 2. Methods

### 2.1. Objectives

The objective of this research is to evaluate the individualized clinical efficacy of TCM in the quantitative way (evidence-based GAS) by long-term observing the variation of symptoms and the corresponding adjustment of prescriptions after the individualized treatment of TCM on NSTE-ACS.

### 2.2. Study Setting and Trial Design

This is observational research which will conduct at the Dongzhimen Hospital Affiliated to Beijing University of Chinese Medicine (BUCM), the Third Affiliated Hospital of BUCM, and the Beijing Hospital of Traditional Chinese Medicine Affiliated to Capital Medical University. This research has a prospective feature, which means that the admission period of patients will be between March 1, 2020, and August 31, 2020. We will include 200 hospitalized patients who were diagnosed as NSTE-ACS. Each included patient will be followed up by telephone or door visit every 2 weeks, collecting the individualized intervention of TCM and evaluating through the table of evidence-based GAS. The whole procedure of the research will consist of 6 times of follow-up. This protocol conforms to the statement of Strengthening the Reporting of Observational Studies in Epidemiology (STROBE) [11] (see Supplementary File 2) and is made some adjustments based on the adaptability for TCM.

### 2.3. Study Participants

#### 2.3.1. Diagnostic Criteria

1. *Diagnostic Criteria of NSTE-ACS for Western Medicine*. The criteria are based on the 2014 AHA/ACC Guideline for the Management of Patients with Non-ST-Elevation Acute Coronary Syndromes [12]. (a) Clinical symptom: paroxysmal pain in the retrosternal area and can radiate to either both arms, the neck, or the jaw; intermittent or sustainable symptoms of diaphoresis, dyspnea, nausea, abdominal pain, or syncope; unexplained new-onset or increased exertional dyspnea. (b) Physical examination: the emerging of pulmonary rales or S3 might represent cardiac insufficiency due to myocardial ischemia in high-risk patients. (c) Electrocardiogram: ST depression, transient ST elevation, or new T-wave inversion are the changes in ECG in patients with NSTE-ACS. The ECG can be relatively normal or initially nondiagnostic. (d) Biomarkers of myocardial necrosis: cardiac troponins can indicate a myocardial injury and provide information on risk stratification when they rise within a few hours of symptom onset and remain elevated for several days and exceed the 99th percentile of the reference value at least once. (e) Imaging: echocardiography can show the decrease of left ventricular ejection fraction (LVEF) and the decrease or disappearance of myocardial segmental motion. Cardiac magnetic resonance imaging (MRI), myocardial perfusion imaging, and multisource computed tomography (CT) to obtain the value in the diagnosis and exclusion of NSTE-ACS.
2. *Diagnostic Criteria of NSTE-ACS for TCM*. The criteria are based on the diagnostic criteria of Xiong Bi which come from the diagnostic and effective criteria of TCM [13]. (a) Feeling of precordium pain and can radiate to either or both arms, the neck, or the jaw with intermittent or sustainable characteristics. Patients can be accompanied by palpitations, shortness of breath, sweat, or even dyspnea. (b) Chest pain can be alleviated from a few seconds to tens of minutes. Severe pain can combine with sweat, cold in the limb, pale in the face, cyanosis of the lips and nails, palpitation, or even sudden cardiac death. (c) It can be triggered by overwork, depression, anger, overeating, or catch a cold. (d) Diagnosis can be supported by the examination of ECG, dynamic ECG, treadmill test, or cardiac troponins.

#### 2.3.2. Inclusion and Exclusion Criteria

1. *Inclusion Criteria*. Participants who meet the following criteria will be included. (a) Male or female patients between 18 years old and 85 years old. (b) Conformed to the diagnosis of NSTE-ACS, including unstable angina and non-ST-segment elevation myocardial infarction (NSTEMI), and complied with the diagnosis of Xiong Bi. (c) Had clearly related symptoms of NSTE-ACS or Xiong Bi and the number of episodes of symptoms more than 2 times per week. (d) Time period of hospitalization between March 1, 2020, and August 31, 2020. (e) Agreed to sign the informed consent (see Supplementary File 1).
2. *Exclusion Criteria*. Participants who meet any of the following criteria will be excluded. (a) With a cardiogenic shock, heart rupture, or interventricular perforation. (b) Had the history of major organ surgery within one month along with active or tendency of bleeding. (c) Patients with implantable defibrillators or pacemakers. (d) With severe liver and kidney dysfunction, malignant tumor, or endocrine, urinary, blood system, nervous system, and other serious primary diseases. (e) With cardiovascular diseases including acute pericarditis, subacute infective endocarditis, aortic dissection, and/or severe arrhythmia. (f) With a history of serious allergies or allergic to Chinese or western medicine. (g) Female during the stage of pregnancy and breastfeeding stage. (h) Have participated in other clinical trials.

#### 2.3.3. Criteria of Exit, Discontinuance, and Rejection

Patients will exit based on the situation as follows: (a) physicians will make a decision if patients have allergic reactions, serious adverse events, complications, or special physiological changes and were not suitable for this research or have poor compliance affecting clinical judgment. (b) Patients are unable to continue follow-up and file an application. The discontinuance of research is followed by orders from the sponsor or administrative department. The rejection criteria are based on the inclusion and exclusion criteria, insufficient information, or lost to follow-up.

### 2.4. Interventions

This protocol is the observational setting with no controlled group. The method of self-control is used to compare therapeutic efficiency. All included participants will not apply any particular interventions for the aim of scientific research. The tool of evidence-based GAS is the way of analysis and evaluation, which represents patients who can receive the individualized treatment of TCM or western medicine based on their situations. The only requirement is they should choose the TCM or combined therapy if they have a need to be treated by physicians.

### 2.5. Sample Size

**Figure 1:**
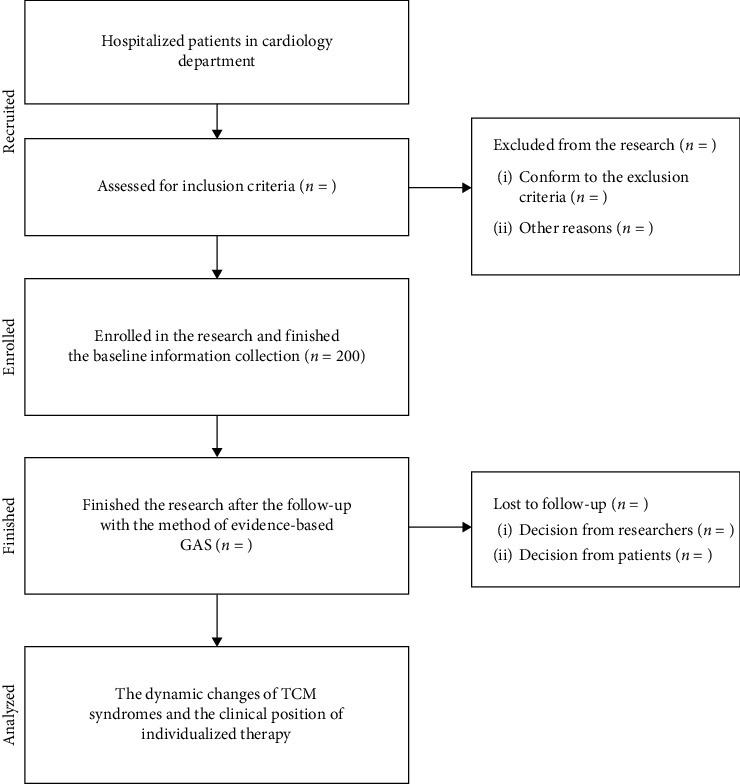
Flow diagram of observational research by the evidence-based Goal Attainment Scale.

This observational research cannot estimate the sample size accurately because of the lack of reference and data support. Two hundred qualified participants will be observed and followed-up according to the support of the foundation, hospitalized reality of the cardiology department previously, and the potential location of NSTE-ACS patients in three hospitals. The materials and methods section should contain sufficient detail, so that all procedures can be repeated. It may be divided into headed subsections if several methods are described. The flow diagram of this research is listed as follows (Figure 1).

### 2.6. Method of Evidence-Based GAS

#### 2.6.1. Importance and Difficulty for the Classification of Weight

This part will be accomplished by patients based on the guidance of researchers. The score of weight that constitutes the evidence-based GAS will be obtained by multiplying the *Importance* times the *Difficulty* (Tables 1 and 2). According to the scoring system, the goal for evaluation will be eliminated if the *Importance* or the *Difficulty* is set as 0. The score of weight is between 1 and 3 points in general. The score of weight will be set as 1 if the physician considers that there is no difference in the score of weight.

#### 2.6.2. Establishment of the Goal of Treatment

The goal of treatment for evidence-based GAS should be consistent with clinical practice. Goals should be set through mutual consultation among physicians, patients, and their families. These goals are people who want to achieve in the course of the treatment. Evidence from the published systematic review and guideline will be used as the support for the establishment of a goal and are listed in Table 2. It is reasonable for most researchers to define the *Baseline* (basic statement before the therapy) at the level of −1 in general unless some symptoms have a greater potential of improvement that can be defined as −2. The formulation of goals should follow the principle named SMART (specific, measurable, achievable, realistic, and timed) which helped physicians shepherd patients to choose realistic goals [14]. Three to five appropriate and reasonable goals will be applied as the basis of evaluation in the final, and the numbers of goals for each individual can be different based on the situation.

#### 2.6.3. Classification of Outcome Scale for Evidence-Based GAS

It should define the classification of outcomes with the 5-level Likert scale after the setting of the goal of treatment to confirm the reliability and validity of results. The related discussion of the classification of outcomes is shown in Section 2.7. The criteria of assignment for every score should be described clearly. The score will be set as -2 if the outcome is far below expectation, −1 a little bit lower than expectation, 0 similar to expectation, 1 a little bit higher than expectation, and 2 higher than expectation. The definition of classification can be a behaviour, a state of emotion, a technique, or a process that reflects the goal. It also can be used as an indicator of progress in achieving the goal.

#### 2.6.4. Expected Results

The expected result of every goal is generally defined as the level 0 for the most likely to occur after treatment based on the 5-level Likert scale (Table 3), and the realization of the expected result will constitute a successful one. The expected result specified by the goal can be as accurate as possible through the detailed setting of every indicator.

#### 2.6.5. Realistic Results

Professional evaluators will assign corresponding scores according to the completion of each goal at the arranged time point after the therapy. *T* score will be calculated by the difference between the score of baseline and result based on the completion of evidence-based GAS. It will be the final score of a patient to evaluate the characteristics of clinical therapy. Two factors should be taken into account when calculating the score: (1) different weight scores (*w*_*i*_) should be given due to the different degrees of each indicator; (2) the influence of quantity variance should be eliminated due to the number difference of each goal. The formula is listed as follows:

(1) $$\begin{array}{c} \text{GAS}=50+\frac{10\sum^{}\left(W_{i}X_{i}\right)}{\sqrt{\left(\left(1-p\right)\sum^{}w_{i}^{2}+p\left(\sum^{}w_{i}^{2}\right)\right)}}. \end{array}$$

Note: *x*_*i*_ represents the score for the indicator of the item *i*; *w*_*i*_ represents the weight for the indicator of the item *i*; *p*=0.3.

### 2.7. Outcomes

#### 2.7.1. Symptoms of Chest Pain

Symptoms of chest pain should be asked by physicians and replenished by family members. Symptoms should be around the frequency of attack, the degree, the duration, the location, the timing, the quality, and the accompanying discomforts which are related to the judgment of TCM syndrome. The discomforts should be on the basis of cold and heat, sweat, head and body, excrement, diet, and so on [15].

#### 2.7.2. Quality of Life

The information of the quality of life comes from the short-form health survey with 36 items (SF-36) [16], which is consisted as follows: (1) assessing physical functioning (PF; 10 items), (2) social functioning (SF; 2 items), (3) role limitation due to physical health (RP; 4 items), (4) bodily pain (BP; 2 items), (5) mental health (MH; 5 items), (6) role limitations due to emotional health (RE; 3 items), (7) vitality (VT; 4 items), (8) general health perceptions (GH; 5 items), and (9) reported health transition (1 item). We choose the item of 1 to 8 for the evaluation of the quality of life.

#### 2.7.3. Situations of Drug Use

The drug contains the application of TCM and western medicine. The situation of drugs for TCM and western medicine will be measured by types, dosage, frequency, duration, and side effects. It should be highlighted that the reasons for the change of drug should be recorded clearly. The application of drugs should follow the medical advice of doctors strictly.

#### 2.7.4. Financial Burden

The financial burden is an essential part of patients [17]. The financial burden of patients will be evaluated from the direct burden, the indirect burden, and the invisible burden [18]. The direct burden will be measured by the payments for health services. The indirect burden will be measured by the loss of social and family values due to the disease. The invisible burden will be measured by the decrease in quality of life due to disease.

#### 2.7.5. Prognosis of Patients

The prognosis of patients will be asked by the report of major adverse cardiovascular events (MACEs) [19], which are listed as follows: (1) cardiovascular cause of death. The attribution of death should be in the subcategories of acute myocardial infarction, sudden cardiac death, heart failure, stroke, cardiovascular procedure, and cardiovascular haemorrhage. (2) Myocardial infarction (MI). Its diagnosis is based on cardiac troponin and the MB fraction of creatine kinase (CK-MB) [20]. (3) Stroke. It is defined on the basis of the presence of acute infarction as demonstrated by imaging or the persistence of symptoms. (4) Transient ischemic attack (TIA). It is the transient episode of focal neurological dysfunction caused by the brain, spinal cord, or retinal ischemia [21]. (5) Percutaneous coronary intervention (PCI). The stents can be the new classification named drug-coated balloons and bioresorbable drug-eluting stents/scaffolds. (6) Peripheral vascular intervention. The concentrated area of it includes the infrarenal aorta, iliac, and infrainguinal arteries and carotid, renal, mesenteric, and aortic interventions. (7) Rehospitalization: this is due to cardiovascular events.

#### 2.7.6. Physical and Chemical Indicators

The indicators including 7 categories will be measured as follows: (1) blood lipid: cholesterol (CHO), triglycerides (TG), high-density lipoprotein (HDL), and low-density lipoprotein (LDL). (2) Vascular endothelial function: C-reactive protein (CRP), nitric oxide (NO), and endothelin (ET). (3) Blood rheologic indexes: erythrocyte sedimentation rate (ESR). (4) Oxidative stress: malondialdehyde (MDA) and superoxide dismutase (SOD). (5) Myocardial injure marker: creatine kinase-MB (CK-MB), creatine kinase (CK), cardiac troponin T (cTnT), cardiac troponin I (cTnI), and lactate dehydrogenase (LDH). (6) Liver and kidney functions: aspartate aminotransferase (AST), alanine aminotransferase (ALT), albumin (ALB), blood urea nitrogen (BUN), and serum creatinine (SCR). (7) Blood coagulation factor: prothrombin time (PT), activated partial thromboplastin time (APTT), thrombin time (TT), and fibrinogen (FIB).

#### 2.7.7. Imaging Indexes

The indexes will be collected and inquired including echocardiography (left ventricular ejection fraction, LVEF), chest radiography, chest tomography (CT), and magnetic resonance imaging (MRI).

#### 2.7.8. Efficiency of TCM Syndrome

The efficiency will be evaluated on the basis of the degree of TCM syndrome improvement before and after therapy [13]. The corresponding adjustment is conducted to adopt the 5-level Likert scale of evidence-based GAS. The standard table of TCM syndrome score is calculated as follows: decrement rate of syndrome score (efficacy index %) = [(score of pretreatment − score of posttreatment)/score of pretreatment] × 100%. Enormous amelioration represents the percentage reduction of syndrome score ≥ 90%; obvious amelioration represents the percentage reduction of syndrome score ≥ 70% and ＜90%; a little bit amelioration represents the percentage reduction of syndrome score ≥ 30% and ＜70%; no obvious variation represents the percentage reduction of syndrome score < 30%; obvious aggravation represents the percentage reduction of syndrome score < 0 (Table 3).

### 2.8. Adverse Events

#### 2.8.1. Definition of Adverse Events

This definition contains the appearance or deterioration of any syndrome, symptom, or disease that may affect the health of patients during the observation period. The adverse events can be new diseases, deterioration of symptoms, treatment, or disease, and a combination of one or more factors, which does not represent the direct causal relationship with the individualized treatment of TCM.

#### 2.8.2. Serious Adverse Events

These events include death, life-threatening events, prolonged hospitalization, disability, congenital malformation, crucial medical events (events that may not immediately endanger life or result in death or hospitalization but may endanger the patient or require action to prevent consequences mentioned above), and medical treatment to prevent permanent damage.

#### 2.8.3. Judgment between Adverse Events and Drugs

(1) Whether there is a reasonable relationship between the time of TCM individualized treatment and suspicious adverse reactions. (2) Whether the suspected adverse reactions are consistent with types of adverse events that are already known for TCM individualized treatment. (3) Whether suspected adverse reactions can be reduced and disappear on the basis of drug withdrawal and dosage reductions. (4) Whether the same reaction occurs again after using the same drug. (5) Whether the suspected adverse reactions can be explained by the pathological status, combination of drugs, and previous therapy of patients.

#### 2.8.4. Management of Adverse Event Researchers

The variation of the condition of patients should be recorded for individualized TCM treatment faithfully. Adverse reactions or unexpected toxic side effects (including symptoms, signs, and laboratory indicators) should be paid attention during the evaluation of the efficacy with the evidence-based GAS. The symptoms, degree, occurrence time, duration, and measures of adverse reactions during hospitalization and follow-up should be recorded in the questionnaire and marked with the date.

### 2.9. Statistical Analysis

The software SPSS version 19.0 will be used for statistical analysis. All statistical analysis will be in the significance level 0.05 and expressed by the 95% confidence interval (CI) or two-sided test. The description of quantitative indicators for baseline and the demographic feature will calculate the mean, standard deviation, median (distributed data), and maximum (distribution data). The description of qualitative indicators will calculate the frequency and percentage. Results of evidence-based GAS on different times will be analysed by the repeated measures ANOVA to observe the dynamic characteristics of individualized curative effect. Among the repeated measures ANOVA, the multivariable test (Pillai's trace, Wilks' lambda, Hotelling's trace, and Roy's largest root) will be used to judge the difference of efficiency at different time points; Mauchly's test of sphericity will be applied to determine whether the corrected test (Greenhouse–Geisser, Huynh–Feldt, and lower-bound) is needed; tests of within-subjects effects will be judged whether the result is consistent with the multivariable test; tests of within-subjects contrasts will evaluate whether the change of curative effect was in accordance with the form of the curve; tests of between-subjects effects will be used to determine whether the efficacy of pairwise comparison at each time point was statistically significant.

### 2.10. Quality Control

All researchers will be received the train of protocol and method of evidence-based GAS. All physicians should conduct the protocol according to the standard operating procedure (SOP) in the future.

#### 2.10.1. Selection Bias

(1) Admission bias (Berkson's bias) [22]: the bias due to different admission rates of patients with different diseases in hospital-based researches. (2) Nonresponse bias [23]: the bias due to uncooperative of patients or unwilling to participate, lowering down the response rate. (3) Follow-up bias [24]: patients do not obey the observational plan during the follow-up period. (4) Assembly bias [24]: inconsistencies between factors of this research and factors besides this research that influence the outcome of the disease. (5) Exclusive bias: the false association between factors and disease resulted from the direct exclusion of some participants without strict selection according to the principle of equivalence. As for the method of control, the preventive measures such as restriction, matching, and randomization will be done to prevent confounding factors in the stage of study design; detailed data collection of potential confounding factors will be done in the stage of implantation; various measures of standardization or multivariate analysis will be done in the stage of analysis.

#### 2.10.2. Informational Bias

(1) Recall bias: the systematic bias due to the variation of accuracy and integrity when patients recall their previous therapy. (2) Reporting bias: patients are deliberately exaggerating or narrowing some information. (3) Detection bias: the collection of inaccurate information due to incomplete records and the error analysis. As for the method of control, detailed data collection and strict quality control methods will be developed; the information will be collected by objective indicators or methods; questionnaires should be designed strictly, and researchers should remain scientific [25].

#### 2.10.3. Confounding Bias

This bias will be controlled by (1) preventing confounding factors with restriction and match; (2) collecting detailed and accurate data that may include confounding factors; and (3) taking various measures in the stage of analysis with the methods of standardization and multivariate analysis.

### 2.11. Data Management and Monitor

#### 2.11.1. Data Management

Every participant should follow the instruction during the observational period, and their related information should be recorded and stored as a paper document (CRF). The electrical document should be stored in SPSS along with the procedure of filling in the paper document. All basic information of participants will be protected by the researching group, and a unique identified number will be applied during the procedure of follow-up. The informed consent will be stored in the office of Dongzhimen Hospital of Beijing University of Chinese Medicine along with the CRF.

#### 2.11.2. Monitor

A researcher from the researching group will be assigned as the clinical research coordinator (CRC) to assist experts in nonmedical judgment related affairs. CRC will compare the data between the paper and electrical document, and any modification of the data will be monitored and recorded in detail.

## 3. Discussion

The evaluation of clinical efficiency for modern western medicine paid close attention to the variation of laboratory indexes or pathohistological structure [26]. The evidence derives from objective and authentic results of human trials with rigorously designed, such as randomized controlled trials (RCTs) [27]. The distinguishing feature of dynamic, complicated, and multitarget effects for TCM cannot be reflected fully in the current evaluating system and model [10]. Clinical research of TCM should combine with the theory of evidence-based medicine, conducting the design and progress on the basis of syndrome differentiation of TCM and elucidating the result according to the element of TCM [28]. Evidence-based GAS is a method of evaluation combined with GAS and evidence-based medicine, which has the ability to objectively measure the individualized efficiency of TCM.

There are some obvious advantages of evidence-based GAS on the clinical application of TCM. Firstly, mutual communication in-depth between patients and physicians will be done to assist the construction of much more rational plan of TCM. Secondly, personal clinical experience and objective clinical evidence will be well combined to better show the character of TCM and promote to the whole world. Thirdly, this method primarily focuses on the dynamic and quantitative evaluation for TCM personalized treatment, which is much more suitable for TCM than standardized grouping evaluation.

Limitations for evidence-based GAS on clinical practice for observational research are listed as follows: (1) evidence-based GAS comes from the optimization of GAS, in which the process of evaluation is conducted according to the personalized intention of patients. Although evidence-based GAS combined with the evidence-based medicine of TCM and western medicine, the practicability and acceptability of it for personalized evaluation for TCM is still in at the centre of controversy; (2) working efficiency of researchers will be influenced on the basis of long-term observation and follow-up, and some of the events such as recall bias and follow-up bias cannot deal with appropriately; (3) although the clinical evidence from this observational study design can reflect the characteristics of individualized therapy for TCM, the evidence is still in a low level (II-3 level of the US Preventive Services Task Force [29]; C level of the Oxford Centre for Evidence-Based Medicine [30]) which needs further demonstration from experts.

## Figures and Tables

**Table 1:** The classification of score of weight for *Importance* and *Difficulty*.

Importance	Difficulty
0 = not important	0 = not difficult
1 = slightly important	1 = slightly difficult
2 = important	2 = difficult
3 = very important	3 = very difficult

**Table 2:** The goal of treatment from physicians and evidence-based medicine (3 to 5 goals among the 8 goals can be selected form the table).

Goal of treatment	Importance	Difficulty	Weight	Baseline	Results
1. Whether can improve the symptom of chest pain					
2. Whether can improve the quality of life (based on the SF-36 form)					
3. Whether can reduce the drug use					
4. Whether can reduce finical burden of patients					
5. Whether can improve prognosis of patients based on the report of MACE					
6. Whether can improve the physical-chemical indexes (blood lipids, vascular endothelial function, blood rheologic indexes, oxidative stress, blood routine, liver and kidney functions, and blood coagulation factor)					
7. Whether can improve imaging indexes (LVEF, chest radiography, CT, and MRI)					
8. Whether can improve syndrome efficiency of TCM					
9. Handwritten: the first goal of treatment that patients they want					
10. Handwritten: the second goal of treatment that patients they want					
*Sum of weight*					

**Table 3:** Scale for evidence-based GAS.

Scale for evidence-based GAS	−2	−1	0	1	2
	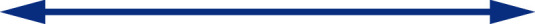				
1. Symptom of chest pain	Increase of the frequency of attack and the degree of pain, prolong for duration	No change of the frequency of attack, the degree of pain, and duration	A little bit relieves of the frequency of attack, the degree of pain, and duration	Obvious relieves of the frequency of attack, the degree of pain, and the duration	No any symptoms of chest pain

2. Quality of life based on SF-36 (^1^physiological function, ^2^physical function, ^3^body pain, ^4^general health, ^5^vitality, ^6^social function, ^7^emotional function, and ^8^mental health)	Aggravate obviously for 2 or more among the 8 dimensions of SF-36	No clearly improvement among the 8 dimensions of SF-36	Obvious amelioration for 1 to 2 among the 8 dimensions of SF-36	Obvious amelioration for 3 to 4 among the 8 dimensions of SF-36	Obvious amelioration for more than 4 among the 8 dimensions of SF-36

3. Situations of drug use	Obvious increase of dosage and categories	No obvious variations for dosage and categories	Decrease of dosage and categories (1 to 2)	Obvious decrease of dosage and categories (more than 2)	Discontinue drug followed by the doctor's advice

4. Financial burden	Obvious increase of financial input	No obvious change of financial input compared with before	Decrease of financial input and relieve of the burden	Obvious decrease of financial input and relieve of the burden	No financial burden

5. Prognosis of patients (MACE: ^1^death, ^2^MI, ^3^stroke, ^4^TIA, ^5^PCI, ^6^peripheral vascular intervention, and ^7^readmission)	1 or more new events (MACE) report compared with before	Events were observed similar to before and no new events report	No event (MACE) reports	No event (MACE) reports for a long time (six months to one year)	No event (MACE) reports for a long time (more than one year)

6. Physical and chemical indicators (^1^blood lipid, ^2^vascular endothelial function, ^3^blood rheologic indexes, ^4^oxidative stress, ^5^myocardial injure marker, ^6^liver and kidney functions, and ^7^blood coagulation factor)	Obvious aggravated more than 2 indicators	No obvious change compared with before	Amelioration of 1 to 2 indicators compared with before	Amelioration of 3 to 4 indicators compared with before	Amelioration of more than 4 indicators compared with before

7. Imaging indexes (^1^LVEF, ^2^chest radiography, ^3^CT, and ^4^MRI)	Obvious aggravated more than 2 indexes	No obvious change compared with before	Amelioration of 1 to 2 indexes compared with before	Amelioration of 2 to 3indicators compared with before	Amelioration of more than 3 indicators compared with before

8. Efficiency of TCM syndrome [13]	Obvious aggravated of the TCM syndrome compared with before	No obvious variation of the TCM syndrome compared with before	A little bit amelioration of the TCM syndrome compared with before	Obvious amelioration of TCM syndrome compared with before	Enormous amelioration of TCM syndrome compared with before

9. Handwriting: the goal 1 that patients want to achieve)					

10. Handwriting: the goal 2 that patients want to achieve					

## Data Availability

No data were used to support this study.

## Supplementary Materials

Supplementary Materials Supplementary File 1: informed consent form. Supplementary File 2: checklist of STROBE. Supplementary File 3: document of ethical approval.
