# Supplementary material for: Individualized Efficiency of Traditional Chinese Medicine for Non-ST Segment Elevation Acute Coronary Syndrome: Study Protocol for Observational Research by the Evidence-Based Goal Attainment Scale
Source: Evid Based Complement Alternat Med. 2020 Sep 14;2020:7653040. doi: 10.1155/2020/7653040 (PMC7509552; doi:10.1155/2020/7653040)
Supplement: Supplementary Materials — Supplementary File 1: informed consent form. Supplementary File 2: checklist of STROBE. Supplementary File 3: document of ethical approval. [file 7653040.f1.zip › 7653040.f1/Supplementary File 1 Informed Content.docx]

**Informed Consent**

——*Informed page*——

Dear Sir or Madam：

You will be invited to join a clinical research named “Individualized Efficiency of Traditional Chinese Medicine for Non-ST Segment Elevation Acute Coronary Syndrome by the Evidence-based Goal Attainment Scale”. This research is used to evaluate the clinical efficiency and safety of individualized treatment TCM for non-st segment elevation acute coronary syndrome in long-term period.

Please read carefully before you decide to join this research. The information can help you understand the objective, procedure, duration, benefit, risk and discomfort of the research. You can discuss with your relatives and friends, or require physicians provide explanations, helping you make decision.

1. ***Basic Introduction***
   1. Name: individualized efficiency of traditional Chinese medicine for non-ST segment elevation acute coronary syndrome (NSTE-ACS) by the evidence-based goal attainment scale

Researchers: medical doctor Zhaofeng Shi, medical master Manke Guan, and Xuxu Wei.

Sponsor: Dr. Zhaofeng Shi.

Version and date: version: V1.0, Date: 2020. 01.15

- 1. You will join a perspective and observational research
  2. Objective: evaluate the individualized efficiency of TCM with quantitative way through the long-term observation on the variation of TCM syndrome and corresponding adjustment of drugs after the treatment of TCM on NSTE-ACS.
  3. Process: observational method will be adopted to design the model. Two hundred hospitalized patients diagnosed with NSTE-ACS and conform to the inclusion criteria will be included between March 1, 2020, and July 31, 2020. The evidence-based goal attainment scale (GAS) will be used to evaluation during the follow-up period every two weeks. Individualized intervention of TCM will be recorded in detail for three months. The variation of clinical efficiency and quality of life before and after the treatment will be measured by long-term evaluation, guiding the physical rehabilitation and future therapy of patients.
  4. Duration and deadline: telephone or visit at home will be conducted from 8:00 to 18:00 during the date of follow-up. Deadline will be the date after 3 months since the enrollment of patients.
  5. Times of follow-up: six

Procedure of follow-up: researcher will contact you with telephone, informing the requirement and appointing the time with you. You should prepare the medical record, information of medication and related data of examination for nearly 2 weeks in advance. It is better to have relatives who are familiar with your illness beside you and you need to cooperate with researchers to collect, arrange, and record the data of follow-up. You need to keep your personal medical records properly after the follow-up.

Content of follow-up: treatment of Chinese or western medicine (including names, frequency, and period); major symptoms that bother you; examination of physical, chemical, and imaging; quality of life (SF-36); Major adverse cardiovascular event [MACE, including cardiovascular cause of death, myocardial infarction (MI), stroke, transient ischemic attack (TIA), percutaneous coronary intervention (PCI), peripheral vascular intervention, rehospitalization].

- 1. Inclusion criteria: (a) Male or female patients between 18 years old and 85 years old. (b) Conformed to the diagnosis of NSTE-ACS, including unstable angina and non-ST-segment elevation myocardial infarction (NSTEMI), and complied with the diagnosis of *Xiong Bi*. (c) Had clearly related symptoms of NSTE-ACS or *Xiong Bi* and the number of episodes of symptoms more than 2 times per week. (d) Time period of Hospitalization between March 1, 2020 and August 31, 2020. (e) Agreed to sign the informed consent.
  2. Exclusion criteria: (a) With cardiogenic shock, heart rupture, or interventricular perforation. (b) Had the history of major organ surgery within one month along with active or tendency of bleeding. (c) Patients with implantable defibrillators or pacemakers. (d) With severe liver and kidney dysfunction, malignant tumor, or with endocrine, urinary, blood system, nervous system, and other serious primary diseases. (e) With cardiovascular diseases including acute pericarditis, subacute infective endocarditis, aortic dissection, and/or severe arrhythmia. (f) With a history of serious allergies or allergic to Chinese or western medicine. (g) Female during the stage of pregnancy and breast-feed stage. (h) Have participated in other clinical trial.
  3. Sample size and grouping information

Two hundred qualified participants will be observed and followed-up according to the support of the foundation, hospitalized reality of the cardiology department in previously, and the potential location of NSTE-ACS patients in Dongzhimen hospital of BUCM, Third affiliated hospital of BUCM, and Beijing hospital of traditional Chinese medicine. This study is an observational research, and the comparison is self-control method. Therefore, only the observational group is required.

1. ***Briefly Describe the Research Institution and Qualifications of Researchers***

This project is supported by the key laboratory of Chinese internal medicine of Dongzhimen hospital and cardiovascular department of BUCM. This research is also supported by the National Science Fund for Distinguished Young Scholars (no.81725024). Dr. Zhaofeng Shi, Jiayuan Hu, Min Li, Manke Guan, and Xuxu Wei have been engaged in clinical evaluation and evidence-based medicine of cardiovascular disease of TCM for a long time. They have rich clinical experience. This research is also guided and supported by professor Hongcai Shang, the vice president of Dongzhimen hospital, having the clinical scientific nature and feasibility.

1. ***Explain the Possible Benefits of Participation***
   1. Benefits for social groups: providing evidence to support the clinical efficacy of individualized TCM treatment for NSTE-ACS, and reflecting the effect of individualized TCM intervention on patients over a long-term of time comprehensively.
   2. Benefits for patients: optimizing the application of individualized programs for patients with NSTE-ACS, preventing and reducing the occurrence of cardiovascular end-point events in patients.
2. ***The Discomfort and Risk or the Research for Patients***
   1. Discomfort caused by the medicine or devices: this study is an observational study, and you will not suffer discomfort caused by the medicine or devices because you do not use them for scientific research.
   2. Possible risks of participation: this study is an observational study. You will be observed your medication status of traditional Chinese and western medicine dynamically during the follow-up period. We will not use the medicine or operate the device, and you will not be exposed to the risks caused by the experiment, and the research team will protect your privacy strictly.
3. ***Clarify the Contingency Plan in Case of Emergency in the Course of Research***

Physicians are obliged to give the medical guidance or psychological counseling and assist the family of patients to transfer to the nearest hospital in case of sudden changes in the patient’s condition or emotional fluctuations during the follow-up period.

1. ***Optional Alternative Treatment for the Disease***

None.

1. ***The Costs Associated with Participation***

Self-paying items: none.

Free program: consultation and guidance for rehabilitation from the physician during the follow-up.

1. ***Confidentiality of Research***

All information about you, including the identity, medical history, situation of disease, medical examination, and laboratory indexes, will be kept strictly in the extent permitted by law. The investigator, sponsor, supervisor, members of ethics committee and the national food and drug administration are permitted to access your medical records to verify the authenticity and accuracy of the collected data. It should highlight that our name will not appear in any public information or reports related to this research.

1. ***Statement of the Right for Patients***

The participation of you is entirely voluntary, and you have the right to withdraw from the study at any stage without any penalty or loss of profit, nor will infect the treatment. If you decide not to participated in the study or want to withdraw form the research after the begin of observation, please contact your physician immediately.

Your doctor might terminate this study without your consent:

A. For your condition

B. You failed to follow the regulations of the study or failed to follow up according to requirement.

C. Termination of study.

1. ***Management of Complaints from Patients***

If you have any complaints about participating in the study, please contact the hospital ethics committee at 010-849012709.

**Informed Consent**

——*Consent page*——

I have read the Informed Page carefully and fully understood the purpose, content, method, and possible benefits and risks of participation. The doctor has explained the relevant medical terms clearly, and all my requirements have been solved with clearly illustrations. I understand that I can refuse to join the research or withdraw from the research at any time or any circumstances without the influence of right and previous treatment.

My participation in this study is completely voluntary, and I have given full consideration to it. I have understood the therapeutic effect of the medicine and possible risks during the follow-up period, and have obtained complete and trustworthy information related o this research. I fully understand this clinical study. I volunteer to participate in this research without any pressure and based on free choice. I will cooperate with researchers to receive follow-up visits according to requirement to complete this study.

I agree to have my medical records reviewed by inspectors, clinical researcher and supervisors of the state food and drug administration when necessary.

I will obtain a signed and dated copy of the informed consent.

Signature of patient: Date:

Signature of relationship with the patient:

Telephone numbers:

*Statement of researcher*

I declare that I have explained the contents, procedures, possible risks and benefits of this study to the participants in detail, and provided adequate answers to any questions raised by patient, who has provided a satisfactory response and understanding.

Signature of physician: Date:

Telephone numbers:
