# Supplementary material for: Individualized Efficiency of Traditional Chinese Medicine for Non-ST Segment Elevation Acute Coronary Syndrome: Study Protocol for Observational Research by the Evidence-Based Goal Attainment Scale
Source: Evid Based Complement Alternat Med. 2020 Sep 14;2020:7653040. doi: 10.1155/2020/7653040 (PMC7509552; doi:10.1155/2020/7653040)
Supplement: Supplementary Materials — Supplementary File 1: informed consent form. Supplementary File 2: checklist of STROBE. Supplementary File 3: document of ethical approval. [file 7653040.f1.zip › 7653040.f1/Supplementary File 3 Document of Ethical Approval.pdf]

北京中医药大学东直门医院医学伦理委员会

Ethics Committee of Dongzhimen Hospital Affiliated to Beijing University of Chinese Medicine

伦理审查批件

Approval Notice Template

项目编号：DZMEC-KY-2020-07

项目名称：基于循证目标成就评量法评价非 ST 段抬高型急性冠脉综合征中医个体化疗效

主要研究者：石兆峰	项目类别：科研课题
申办单位：北京中医药大学东直门医院	合同研究组织（CRO）：
审查类别：复审	审查方式：■快速审查□会议审查
审查委员：王蓬文	审查日期：2020 年 05 月 07 日

批准文件（注明版本号及日期）：

1.修正的临床研究方案 （版本号：2.0，版本日期：2020 年 04 月 29 日）

2.修正的知情同意书 （版本号：2.0，版本日期：2020 年 04 月 29 日）

3.任务书/合同书/试验方案 （版本号：1.0，版本日期：2020 年 01 月 15 日）

4.知情同意书 （版本号：1.0，版本日期：2020 年 01 月 15 日）

5.病例报告 （版本号：1.0，版本日期：2020 年 01 月 15 日）

6.主要研究者/课题负责人履历（签名、注明日期）

7.GCP 培训证书

8.利益冲突研究者声明

9.专家论证书 （版本号：1.0，版本日期：2020 年 01 月 15 日）

审查依据：根据 CFDA《药物临床试验质量管理规范》（2003 年）、《医疗器械临床试验质量管理规范》（2016 年）、世界医学会《赫尔辛基宣言》（2013 年）、国际医学科学组织委员会《人体生物医学研究国际伦理指南》（2016 年）、国家卫生计生委《涉及人的生物医学研究伦理审查办法》（第 11 号，2016）等。

审查决定：同意

注：

1、本批件自签发日期有效期 **12** 个月，起止时间 2020-05-07～2021-05-06 ，研究者必须严格使用经审查同意的知情同意书和研究方案。

2、研究开始前，请申请人完成临床试验注册。

3、凡涉及中国人类遗传资源、需要报批的研究项目，需告知在获得中国人类遗传资源管理办公室批准后才能开始。

4、研究过程中若变更主要研究者，对临床研究方案、知情同意书、招募材料等的任何修改，请申请人提交修正案审查申请。

5、发生严重不良事件，请申请人及时提交严重不良事件报告。

6、请按照伦理委员会规定的年度/定期跟踪审查频率，申请人在截止日期前 1 个月提交研究进展报告。

7、研究纳入了不符合纳入标准或符合排除标准的受试者，符合中止试验而未让受试者退出研究。给予错误治疗或剂量，给予方案禁止的合并用药等没有遵从方案开展研究的情况；或可能对受试者的权益/健康以及研究的科学性造成不良影响等违背 GCP 原则的情况，请申办者/监查员/研究者提交违背方案报告。

8、申请人暂停或提前终止临床研究，请及时提交暂停/终止研究报告。

9、完成临床研究，请申请人提交结题报告。

主任委员☑副主任委员☐签字：　　　　　　　　　　时　间：2020.5.7

北京中医药大学东直门医院医学伦理委员会　　　　会议地点：北京中医药大学东直门医院

本项目持续审查频率☐3 个月☐6 个月■12 个月　　　　联系人：商建伟（010）84012709
